# LDH and PDH Activities in the Ischemic Brain and the Effect of Reperfusion—An Ex Vivo MR Study in Rat Brain Slices Using Hyperpolarized [1-^13^C]Pyruvate

**DOI:** 10.3390/metabo11040210

**Published:** 2021-03-30

**Authors:** Gal Sapir, David Shaul, Naama Lev-Cohain, Jacob Sosna, Moshe J. Gomori, Rachel Katz-Brull

**Affiliations:** 1 Department of Radiology, Hadassah Medical Organization and Faculty of Medicine, Hebrew University of Jerusalem, Jerusalem 9112001, Israel; gal.sapir1@mail.huji.ac.il (G.S.); david.shaul@mail.huji.ac.il (D.S.); naamal@hadassah.org.il (N.L.-C.); jacobs@hadassah.org.il (J.S.); gomori@hadassah.org.il (M.J.G.); 2 The Wohl Institute for Translational Medicine, Jerusalem 9112001, Israel

**Keywords:** ischemic stroke, reperfusion, lactate dehydrogenase, pyruvate dehydrogenase, dissolution dynamic nuclear polarization, brain slices

## Abstract

Ischemic stroke is a leading cause for neurologic disability worldwide, for which reperfusion is the only available treatment. Neuroimaging in stroke guides treatment, and therefore determines the clinical outcome. However, there are currently no imaging biomarkers for the status of the ischemic brain tissue. Such biomarkers could potentially be useful for guiding treatment in patients presenting with ischemic stroke. Hyperpolarized ^13^C MR of [1-^13^C]pyruvate is a clinically translatable method used to characterize tissue metabolism non-invasively in a relevant timescale. The aim of this study was to utilize hyperpolarized [1-^13^C]pyruvate to investigate the metabolic consequences of an ischemic insult immediately during reperfusion and upon recovery of the brain tissue. The rates of lactate dehydrogenase (LDH) and pyruvate dehydrogenase (PDH) were quantified by monitoring the rates of [1-^13^C]lactate and [^13^C]bicarbonate production from hyperpolarized [1-^13^C]pyruvate. ^31^P NMR of the perfused brain slices showed that this system is suitable for studying ischemia and recovery following reperfusion. This was indicated by the levels of the high-energy phosphates (tissue viability) and the chemical shift of the inorganic phosphate signal (tissue pH). Acidification, which was observed during the ischemic insult, has returned to baseline level following reperfusion. The LDH/PDH activity ratio increased following ischemia, from 47.0 ± 12.7 in the control group (*n* = 6) to 217.4 ± 121.3 in the ischemia-reperfusion group (*n* = 6). Following the recovery period (*ca.* 1.5 h), this value had returned to its pre-ischemia (baseline) level, suggesting the LDH/PDH enzyme activity ratio may be used as a potential indicator for the status of the ischemic and recovering brain.

## 1. Introduction

Stroke is a leading cause for both death and acquired neurologic disability worldwide [1,2] and the global burden of the disease is increasing. In the US alone the annual incidence of stroke is about 800,000 cases [3]. The primary goal in the management of acute ischemic stroke is the restoration of tissue blood supply by arterial recanalization. There are currently two treatment options to achieve this goal: fibrinolytic agents (such as alteplase or tenecteplase), and mechanical thrombectomy [4]. Today, the management of acute stroke, i.e., the decision on applying these treatment options, is dictated mainly by imaging findings. This practice was endorsed widely and became official in the 2018 American Heart Association/American Stroke Association society guidelines [5]. To indicate that the therapeutic window for this disorder is wider than once believed, the phrase “imaging is brain” [6] was suggested as an update to the iconic “time is brain” coined more than a quarter century ago [7].

Currently, the treatment decision is based mainly on two imaging modalities. Computed tomography (CT) and magnetic resonance imaging (MRI). Between the two, CT is more commonly used to triage patients [4]. However, MR diffusion-weighted imaging (DWI) is more sensitive for the detection of early infarction (while being equivalent in terms of acute hemorrhage detection [8,9]). It has been suggested that MRI can be used as the sole modality for screening patients in centers with sufficient availability [10], using new imaging protocols where the scan times can be reduced to 5 min or less [11,12,13]. However, another study showed that DWI may miss acute stroke in up to 8% of cases when used alone [14]. Even though stroke treatment is currently guided almost exclusively on the basis of neuroimaging, there are currently no imaging modalities that allow determination of tissue viability in the regions defined by current techniques as ischemic penumbra (i.e., viable brain tissue in a state of hypoperfusion, which can still be salvaged by reperfusion strategies). Accordingly, it has become increasingly imperative to develop new diagnostic and prognostic techniques that are able to assess and provide greater information on the vulnerability of the tissue at risk, and better evaluate the effectiveness of treatments.

Slow progress and generally poor translation from in vivo studies in small animal models of stroke to humans is partly related to the inefficiency of animal models for this condition [15]. In vivo models of stroke in rodents result in a small and heterogeneous ischemic penumbra which may be challenging for imaging. Here, we used the perfused rat brain slices model for generating a homogeneously ischemic brain tissue and monitor its response to the ischemic insult and to reperfusion. The advantage of this ex vivo model is that environmental processes can be regulated and monitored. For example, the perfusion can be controlled (to simulate stroke and reperfusion). ^31^P-NMR of the high-energy phosphates and the inorganic phosphate in the slices allowed real-time monitoring of the response to ischemia and recovery in terms of ATP content and pH, respectively. ^31^P-NMR spectroscopy has been previously utilized for studies of cerebral ischemia both ex vivo [16,17] and in vivo in animals [18,19,20], and in humans [21].

^13^C-NMR of hyperpolarized substrate metabolism [22] using the product-selective saturating excitation approach [23] allowed real-time detection of instantaneous changes of in-cell metabolic enzyme activities. The most widely used hyperpolarized agent so far is [1-^13^C]pyruvate and it is the lead agent for clinical use due to its favorable chemical, physical, and biological properties [24,25,26]. Hyperpolarized [1-^13^C]pyruvate has been previously used to observe rapid metabolic processes in many promising applications [27,28,29,30]. Specifically, real-time brain metabolism was investigated in vivo in small animals [31,32,33,34,35,36,37,38,39,40,41], in small animal models related to stroke [42,43], in the human brain [25,44,45], and in the rat brain ex vivo [46].

In the current study hyperpolarized [1-^13^C]pyruvate was used as the metabolic substrate. In the brain, [1-^13^C]pyruvate is metabolized predominantly by the intracellular enzymes lactate dehydrogenase (LDH) and pyruvate dehydrogenase (PDH) [46] to form [1-^13^C]lactate and ^13^CO_2_, respectively, while the latter is rapidly converted by carbonic anhydrase (CA) to [^13^C]bicarbonate. As pyruvate metabolism represents the crossroad between aerobic and anaerobic metabolism, it appeared likely to have the capability to report on the response to ischemia and reperfusion. The overarching goal of this study was to evaluate the dynamic changes in LDH and PDH activities following ischemia and reperfusion, using in parallel ^31^P NMR indicators to validate the ischemic status of the tissue.

## 2. Results

### 2.1. Brain Slices Remain Viable throughout the Experiment

**Figure 1:**
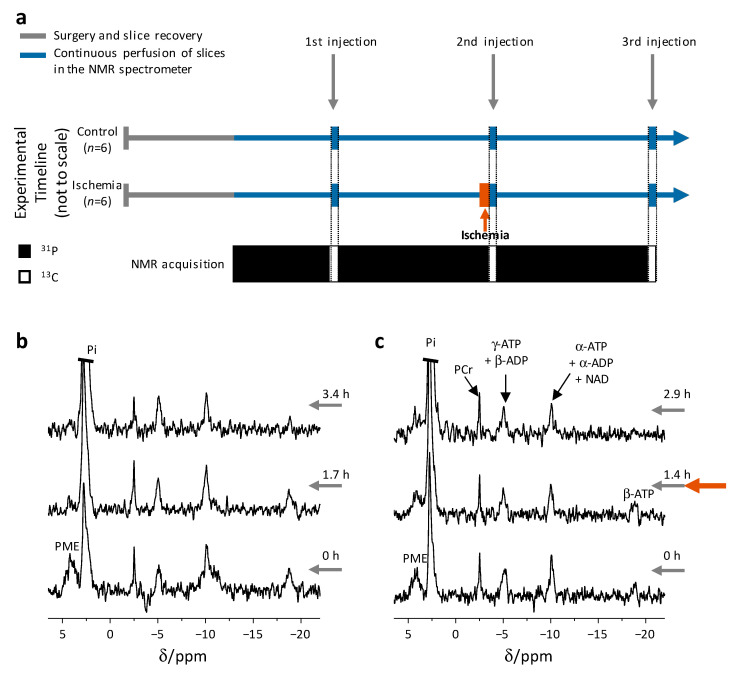
Experimental timeline and slice viability throughout the experiments. (**a**) Experimental workflow. Following surgery and slice recovery (solid grey timeline), the brain slices were placed in the NMR tube and were continuously perfused therein (solid blue timeline). The control group was maintained in this state for the entire duration of the experiment. The 1st injection of hyperpolarized medium was administered about 1 h following the start of perfusion in the NMR spectrometer. About 1.5 h after the 1st injection, the 2nd injection of hyperpolarized medium was administered. For the ischemia-reperfusion group, perfusion was stopped for *ca.* 11 min (brown rectangle) prior to the 2nd injection. A third injection was performed about 1.5 h after the 2nd injection. Injections are marked by grey arrows and the injection times are marked per injection. Injections coincide with ^13^C acquisitions, and ^31^P acquisitions are performed before and after the injections. (**b**) Typical ^31^P NMR spectra acquired from brain slices during continuous perfusion in the spectrometer during a control experiment. (**c**) Typical ^31^P NMR spectra acquired from brain slices in the perfusion system during an experiment with an ischemic insult. The ischemic insult (brown arrow) was applied once the acquisition of the middle ^31^P spectrum was completed. The second injection was administered at the end of the ischemic insult (and provided reperfusion). Times are indicated in h (the time of the first injection of [1-^13^C]pyruvate was referred to as *t* = 0). The acquisition of the ^31^P spectra was completed before each injection (for the second injection, the acquisition was completed before the ischemic insult). Each spectrum was acquired over 1 h, using 3296 excitations (1.1 s repetition time, 50° nutation angle, in batches of 412 scans). The spectra were processed with a line-broadening of 7 Hz and zero-filled from 8,192 to 16,384 points. The spectra were referenced to the PCr signal at −2.5 ppm. The baseline was manually corrected. The signal of Pi was truncated for better visualization of the high-energy phosphates’ signals. The increase in the relative intensity of the Pi signal in the second and third spectra is due to a high phosphate content in the injected hyperpolarized medium which was not fully washed out following the injections. ATP—adenosine triphosphate, ADP—adenosine diphosphate, PCr—phosphocreatine, Pi—inorganic phosphate (intra- and extracellular), PME—phosphomonoesters, NAD—nicotinamide adenine dinucleotide.

Typical ^31^P spectra from both the control and the ischemia-reperfusion groups are shown in Figure 1b,c, respectively. The high-energy phosphate compounds were observed throughout the experiments. Within the ischemia group, the ATP content slightly decreased following the ischemic insult. This decrease can be attributed to loss of tissue viability due to the ischemic insult (Figure S3). For the control group there was no change in the ATP content throughout the experiment. These findings suggested that the slices mostly recovered from the ischemic insult, and that this system may be considered as a model for the ischemic brain and its recovery following reperfusion.

### 2.2. Rapid Changes in the Slices’ pH and Energetic State Revealed by ^31^P NMR Spectroscopy

**Figure 2:**
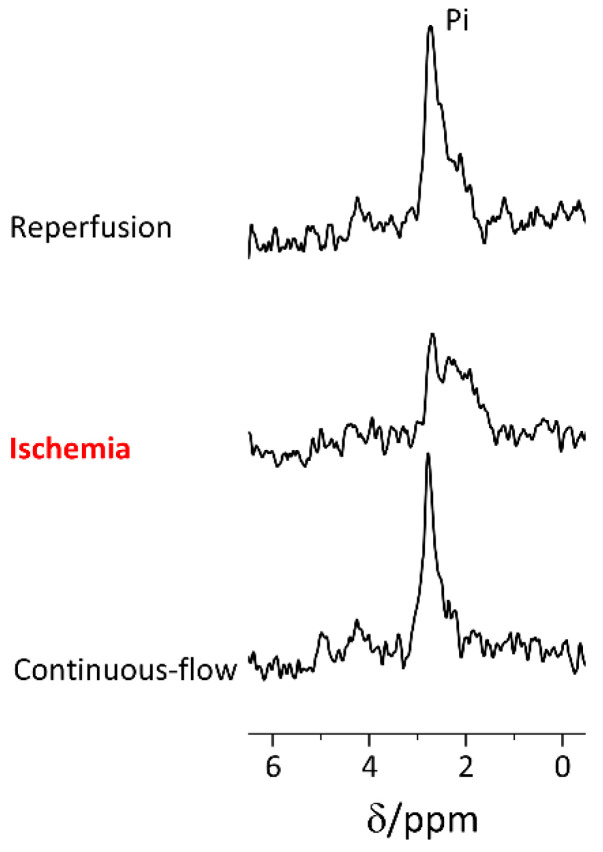
^31^P spectra demonstrating rapid changes in the Pi signal chemical shift and lineshape due to ischemia. The spectral region showing the Pi signal before (bottom), during (middle), and immediately following (top) the ischemic insult in a typical experiment. The Pi signal widens upon ischemia and narrows following reperfusion indicating alterations in pH. The spectra were collected over 7.6 min (412 excitations, 1.1 s repetition time, 50° nutation angle) and processed with a line-broadening of 7 Hz and zero-filling from 8192 to 16,384 points. The chemical shift was referenced to the PCr signal at −2.5 ppm. The baseline was manually corrected. Pi—inorganic phosphate (intra- and extracellular).

During ischemia, short (7.6 min) ^31^P acquisitions were recorded. Figure 2 shows that the signal of the inorganic phosphate in this spectrum is displaced to lower field and its line shape is different. This finding indicated acidification of the tissue and the medium combined, as the chemical shift of the Pi signal can be used as a pH indicator [47,48]. As the perfusion medium contains inorganic phosphate, the Pi signal is composed of both intracellular and extracellular compartments and both appear to acidify during the perfusion arrest. These changes were observed in all of the ischemia-reperfusion experiments (*n* = 6). Taking the extra- and the intracellular compartments signals together as one signal, we analyzed the overall pH change using the multi-parametric approach proposed by Lutz et al. [49] and found a reduction of 0.22 ± 0.12 pH units (weighted mean pH) during the perfusion arrest period, from 7.01 ± 0.09 before the perfusion arrest period to 6.79 ± 0.08 following this period (in 6 experiments, *p* = 0.001, in a paired, two-tailed Student’s *t* test).

The signals of the high energy phosphates were not readily quantifiable in these short acquisitions and showed low signal-to-noise ratio. Using a moving sum approach, we were able to qualitatively show the temporal changes in the PCr and the ATP content of the slices during the brief periods of ischemia and the immediate reperfusion that followed it (Figure S2).

### 2.3. Hyperpolarized [1-^13^C]Pyruvate Metabolism Reveals Reversible Metabolic Changes Following an Ischemic Insult and Reperfusion

**Figure 3:**
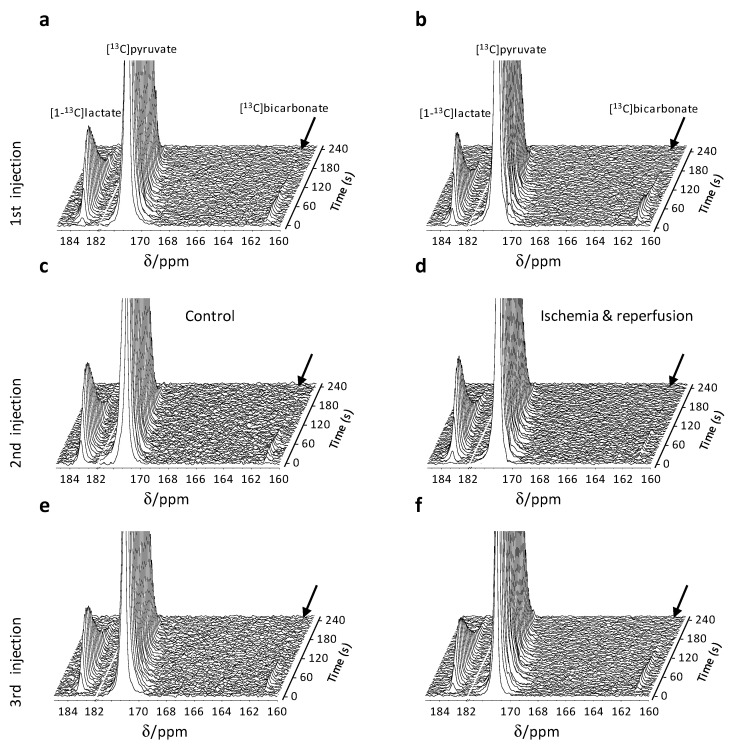
^13^C spectra of hyperpolarized [1-^13^C]pyruvate metabolism in rat brain slices. (**a**–**f**) Stacked ^13^C NMR spectra following injection of hyperpolarized [1-^13^C]pyruvate. (**a**,**b**) Spectra acquired following the first injection in a control experiment and an ischemia and reperfusion experiment, respectively. (**c**,**d**) Spectra acquired following the second injection in a control experiment and an ischemia-reperfusion experiment, respectively. Note the reduced intensity of the [^13^C]bicarbonate signal in d. (**e**,**f**) Spectra acquired following the third injection in a control experiment and an ischemia and reperfusion experiment, respectively. Note the recovery of the [^13^C]bicarbonate signal *ca*. 1.5 h after the ischemia in f. Acquisition parameters: 2.5 ms cardinal-sine frequency-selective pulse, 16,384 time-domain points, 12.5 kHz spectral width. The spectra were acquired in an interleaved manner, with a repetition time (TR) of 4 s between excitations, yielding an 8 s repetition time for each metabolite. The spectra were processed with 5% drift correction, 7 Hz line-broadening, zero-filling from 16,384 to 32,768 points, manual phase and baseline correction, and referenced to the [1-^13^C]pyruvate signal at 171.0 ppm.

Figure 3 shows consecutive ^13^C spectra from typical control (a, c, and e) and ischemia-reperfusion (b, d, and f) experiments. Following the first injection, the signals of the products [1-^13^C]lactate and [^13^C]bicarbonate were observed at similar intensities in both experimental groups (Figure 3a,b). In the second injection, for the ischemia-reperfusion group, the spectra were acquired immediately following ischemia and the hyperpolarized medium actually provided reperfusion to the slices. Figure 3d, shows that in this condition, the [^13^C]bicarbonate signal was greatly reduced, compared with the control experiment (Figure 3c). In the third injection, performed approximately 1.5 h later, the intensity of the hyperpolarized [^13^C]bicarbonate signal recovered, indicating the reversibility of this metabolic change inflicted by the ischemic insult.

### 2.4. Quantitative Analysis of the Enzymatic Activities Demonstrates the Utility of the LDH/PDH Ratio as an Indicator of Ischemia-Reperfusion and Recovery of the Brain

**Figure 4:**
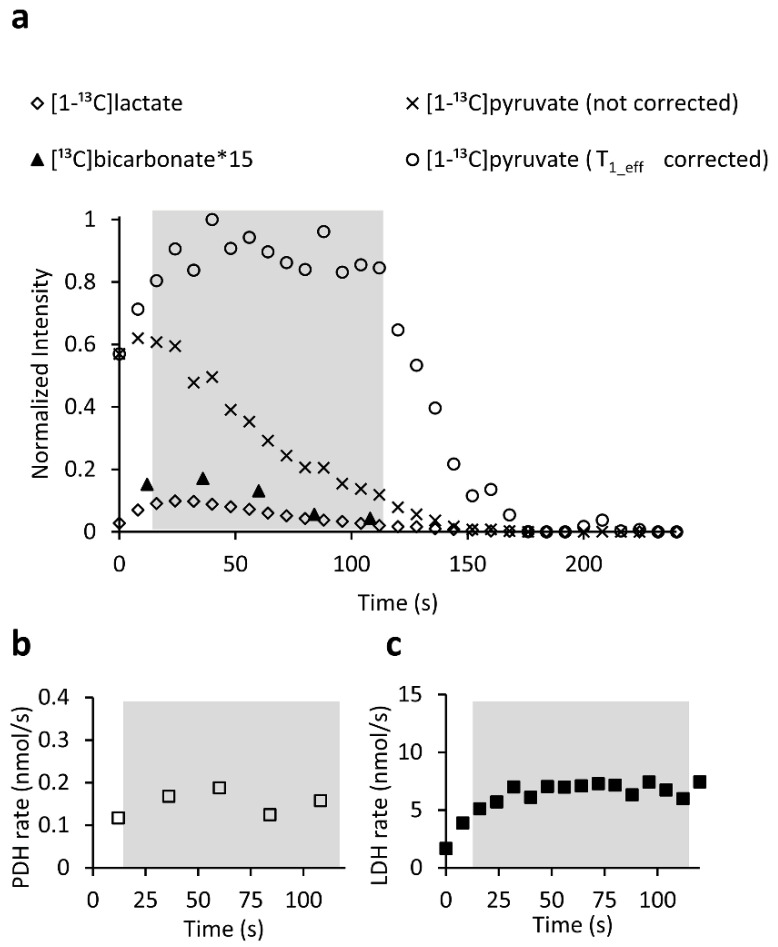
Signal time courses and selection of time points for enzymatic rate determination. (**a**) A typical time course of the hyperpolarized signal intensities following injection of hyperpolarizedpyruvate to brain slices. The measured [1-^13^C]pyruvate signal is shown with “x” markers and the T_1_eff_ corrected signal is marked with open circles. The T_1_eff_ corrected signal was used to determine the time frame at which the concentration of [1-^13^C]pyruvate in the sensitive region of the NMR probe was constant and maximal (shaded area). Only measurements within this time frame were used for the enzymatic rate determination (Methods). For [^13^C]bicarbonate, the averaged spectrum of three consecutive spectra was used for each point. (**b**) The PDH rate determined from the data presented in a. Only points in the shaded area were used for rate calculation. (**c**) The LDH rate determined from the data presented in a. Only points in the shaded area were used for rate calculation.

To quantify the enzymatic rates, the data were acquired with the product selective saturating-excitation approach [23]. Figure 4a shows the signal time course following a typical injection of hyperpolarized [1-^13^C]pyruvate. Only time points for which the concentration of [1-^13^C]pyruvate was constant were used for rate determination. Figure 4b,c show the respective rates of PDH and LDH over time following the injection. Comparing the enzymatic rates in the ischemia reperfusion group, PDH activity was significantly lower in injection 2 compared to either injection 1 or injection 3 (*p* = 0.001 and *p* = 0.05, respectively, in a non-parametric Tukey-type multiple comparison using the Nemenyi test). Following ischemia, the PDH average value was reduced by 72% from its initial value. In the control group there were no differences between the results obtained in the three injections of any of the tested parameters (Kruskal–Wallis single factor analysis of variance by ranks). The entire dataset with enzymatic rates per injection is provided in the Table S5.

**Figure 5:**
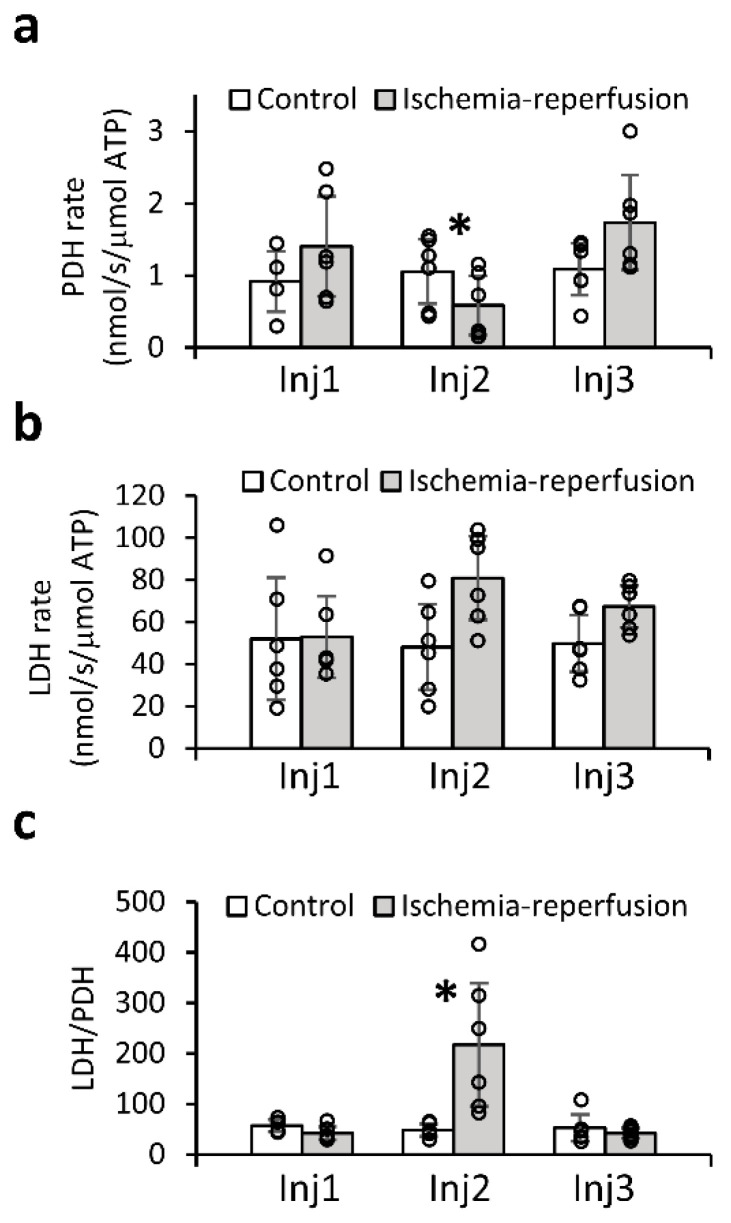
Production rates of [1-^13^C]lactate and [^13^C]bicarbonate. (**a**,**b**) Enzymatic activity relative to the ATP content of the slices (in nmol/s/μmol ATP), for PDH and LDH, respectively. In a, 16 injections were taken in total (*n* = 4 for Inj1, *n* = 6 for each of Inj2 and Inj3). For b, 18 injections are taken in total (*n* = 6 for each of three injections). (**c**) LDH to PDH activities ratio. The enzymatic activities were determined by the production rate of hyperpolarized [1-^13^C]lactate and [^13^C]bicarbonate, respectively, (*n* = 16 injections in total, *n* = 4 for Inj1 and *n* = 6 for each of Inj2 and Inj3). For each group, the ratio was determined per injection (dividing the averaged LDH activity of that injection to the PDH activity determined in the same injection), then averaged per injection number per group. PDH—pyruvate dehydrogenase, LDH—lactate dehydrogenase. Bars and error bars represent the mean and standard deviation, respectively. Circles mark values from individual injections. *—Significant difference.

Figure 5 shows the enzymatic rates of PDH and LDH normalized to the ATP content of the slices. The normalization to the ATP content was performed to account for possible differences in the amount of viable tissue which was involved in the metabolic activity determined on each hyperpolarized injection. This analysis found that the PDH activity was reduced following the ischemic insult compared to its level following recovery (Figure 5a). Additionally, to eliminate the influence of possible confounders related to the quantification of the enzymatic rates (such as amount of tissue and medium in the probe and tissue viability) the ratio of these two enzymatic activities was calculated as well. This calculation showed that the ratio of the LDH and PDH activities increased significantly following ischemia, and returned to the pre-ischemia (i.e., control) level following a 1.5 h recovery period (Figure 5c). On the second injection, the LDH/PDH ratio was 47.0 ± 12.7 (mean ± standard deviation, median = 45.0, interquartile range = 16.5) for the control group and 217.4 ± 121.3 (mean ± standard deviation, median = 196.2, interquartile range = 191.0) for the ischemia group (*p* = 0.005, unpaired two-tailed Student’s *t*-test, *n* = 6). Comparing the LDH/PDH ratio within the ischemia reperfusion group, the ratio was significantly higher in injection 2 compared to either injection 1 or injection 3 (*p* = 0.001 and *p* = 0.01, respectively, in a non-parametric Tukey-type multiple comparison using the Nemenyi test). Within the control group there were no differences in the LDH/PDH ratio obtained on the three injections (Kruskal-Wallis single factor analysis of variance by ranks). Of note, in the control group, the enzymatic activities of both PDH and LDH remained unchanged throughout the 3 injections (Figure 5a,b, respectively). The entire dataset with enzymatic rates per injection is provided in the Supplementary Materials (Table S5).

## 3. Discussion

^31^P spectroscopy was previously used to observe cerebral injury following ischemia and reperfusion and demonstrated two main types of injury: reversible, and irreversible. The former was characterized by a complete or near-complete recovery of the ATP and PCr signals [16,19,50]. For example, in rat hippocampal slices, it was shown that following a 10 min ischemic insult, the high-energy phosphates recovered to ~90% of pre-ischemic levels within 60 min, with ~60% recovery following a 16 min insult [16]. Similar characteristic changes in the ^31^P spectra during- and immediately following ischemia and reperfusion were observed here, suggesting that the current experimental model represents a reversible ischemic brain injury. The clinical correlate of this condition is likely the salvageable brain tissue within the ischemic penumbra of an ischemic stroke.

For the quantification of enzymatic activity rates, we used product-selective saturating excitations [23]. Previously, a ratio between enzymatic activities measured using this method allowed differentiation of benign from malignant tissues [51]. Enzymatic activity ratios are a compelling biomarker, since such ratios are affected primarily by the state of the tissue observed and are independent of the mass of the tissue (i.e., voxel size on imaging and other possifble anatomical based confounders). Using this acquisition approach for ^13^C dDNP NMR spectroscopy we found that the LDH/PDH activity ratio was significantly increased following ischemia, and returned to the pre-ischemic level following the recovery period. This finding suggested a potentially novel metabolic marker of the ischemic penumbra.

^13^C dDNP-MR was previously used in the study of animal models of stroke using other set-ups, hyperpolarized substrates, and acquisition approaches. In one study, the authors used the transient middle cerebral artery occlusion (MCAO) model in mice [43], and observed the conversion of hyperpolarized [1-^13^C]lactate to [1-^13^C]pyruvate and [^13^C]bicarbonate, with an increased [1-^13^C]pyruvate to [1-^13^C]lactate signal ratio 1 h following reperfusion, which returned to baseline levels 2 h after reperfusion. In another study, the authors used a model of endothelin-1 injection (a potent vasoconstrictor) [42] and found that the signals of hyperpolarized [1-^13^C]pyruvate and [1-^13^C]lactate were increased in the ischemic penumbra region 18 h following the induced stroke, while the [1-^13^C]lactate/[1-^13^C]pyruvate signal ratio remained unchanged. Another study examined the effect of ischemia on the developing mouse brain [52], and found that the hyperpolarized [1-^13^C]lactate to [1-^13^C]pyruvate signal ratio was higher in the injured hemisphere compared to the control one 2–5 h following the insult, and returned to baseline levels 20 days following the insult. It is important to note that in addition to the use of other model systems than the set-up used here (such as in vivo vs. ex vivo and different time scales), these previous studies did not determine enzyme activity. Therefore, it is not possible to directly compare the current results to these prior studies. Nevertheless, a possible unifying explanation for these prior observations and the current ones is as follows. The earliest changes following an ischemic insult consist of imbalance in intracellular enzymatic activities as the oxygen content is consumed, leading to the finding presented here (i.e., an increase in the LDH/PDH activity ratio immediately following ischemia). This change may be followed by increased MCT expression and activity (demonstrated as early as 1 h following an ischemic insult [53]), as suggested by Hyacinthe et al. [43]. Later on, blood–brain barrier disruption may have led to the increased amounts of both [1-^13^C]pyruvate and [1-^13^C]lactate, which were observed 18 h following the ischemic insult [42]. Thus, hyperpolarized MR may allow in vivo determination of the state of the brain tissue on the spectrum between healthy, salvageable, and non-salvageable using multiple parameters that can be deduced from hyperpolarized ^13^C spectroscopy such as signal size, signal ratio, and enzyme activities and their ratios.

Ex vivo studies of brain slices are desired as they can be used to ameliorate confounding effects of blood–brain barrier (BBB) transport and tissue blood flow seen in whole animal studies [46,54] and specifically in studies in small animals. Nevertheless, transport across the BBB is part of the in vivo scenario, and may be modulated during ischemia. This may affect the determined metabolic rates in real-time and their effectiveness in reporting on the status of the ischemic penumbra. Ex vivo studies also eliminate the effects of anesthesia, which has been previously shown to alter many aspects of cerebral metabolism and blood flow in general [55], and the levels of hyperpolarized signals following an injection of [1-^13^C]pyruvate specifically [32]. In this regard, ex vivo studies more closely resemble the clinical scenario in which patients undergoing neuroimaging for stroke are not under anesthesia. Additionally, the metabolism observed using this model represents brain parenchyma metabolism alone, without confounding effects of measuring the metabolism in the soft tissues surrounding the brain, metabolites influx from remote tissues, and the metabolites in the cerebral blood vessels [40]. Our findings suggest that the increased LDH/PDH activity ratio observed here is an inherent characteristic of the ischemic brain tissue that can be recovered following reperfusion.

Despite tremendous effort invested in the development of neuroprotective agents, there are currently no FDA-approved treatments for acute ischemic stroke apart from reperfusion strategies [4]. We suggest that the ex vivo model investigated here may provide another avenue to examine the effects of future treatments. We have demonstrated that the effects of an intervention can be observed in real-time and monitored using the LDH/PDH ratio as well as the acidity and the energy status. As the technology is non-destructive, further biochemical assays can be applied to the slices when the experiment is completed. Additionally, we suggest that the LDH/PDH activity ratio demonstrated here warrants further investigation in vivo. If successful, it could potentially serve as a biomarker for neuroimaging studies in patients experiencing ischemic stroke. As such a biomarker is desperately needed, further studies are needed for the validation of this biomarker. In recent years it has been demonstrated that the results of stroke studies in rodents are not fully translatable to humans, likely due to the fact that these small animals have small lissencephalic brains and not large gyrencephalic, human-like brains [56,57], and that stroke in small rodents creates a heterogeneous penumbra. For this purpose, the common pig and miniature pig were suggested as relevant stroke models [56]. It appears warranted to suggest that further in vivo validation of this potential biomarker should be performed in such a large animal model.

### Limitations

Despite the unambiguous findings presented here, this study is not without its limitations. Most importantly, the acquisition strategy used here to quantify enzymatic rates is not yet available for in vivo studies. However, we do note that following injections of hyperpolarized [1-^13^C]pyruvate, both [1-^13^C]lactate and [^13^C]bicarbonate were successfully observed in the human brain in a the relevant timescale [44]. In addition, we note that acquisition strategies that rely on frequency selective excitations have been previously applied in multiple in vivo studies [58,59,60]. Specifically, an excitation scheme in which a 90° excitation was applied to the product site and a low flip angle to the precursor signal, has been used previously by Schulte et al. [60] to determine in vivo a voxel specific metabolic rate from a single time point. Therefore, it is likely that the product-selective saturating excitation acquisition approach that was used here for determining enzymatic rates and their ratio, will be translatable to in vivo and clinical studies. Models for calculating enzymatic rates from in vivo and clinical studies using such acquisitions are likely to be developed. 

We also note that in the whole animal (and in humans), collateral flow is almost always present, and thus the extreme condition of a complete absence of perfusion is rarely encountered. Here, we used a relatively short ischemic period which was induced by halting the perfusion flow. However, we note that the extracellular volume was filled with oxygenated medium prior to perfusion arrest. Although the oxygen level in this medium clearly dropped during the perfusion arrest period (Note S4), it is hard to estimate the actual oxygen availability to the brain cells in the slices during this time. Additionally, ^31^P NMR spectroscopy for the measurement of slice ATP in our experimental setup is not an immediate process (i.e., requires at least 30 min to be completed), and therefore cannot provide and immediate correlate of the oxygenation status. We note that due to the low SNR of ^31^P NMR, here and in the clinical setting, it is not likely that ATP quantification or pH measurement will be relevant in a clinical scenario. We therefore propose the LDH/PDH ratio, which is independent of tissue volume and number of live cells, as a potential robust and rapid indicator of brain tissue status.

## 4. Materials and Methods

### 4.1. Chemicals

The OX063 radical (GE Healthcare, UK) was obtained from Oxford Instruments Molecular Biotools (Oxford, UK). [1-^13^C]pyruvic acid was purchased from Cambridge Isotope Laboratories (Tewksbury, MA, USA). NaCl, KCl, D-glucose, NaHCO_3_, MgSO_4_, NaH_2_PO_4_, N-methyl-D-glucamine (NMDG), and CaCl_2_ were purchased from Sigma-Aldrich, (Rehovot, Israel). Isoflurane was obtained from the Institutional Authority for Biological and Biomedical Models of the Hebrew University (Jerusalem, Israel).

### 4.2. Animals

Female Sprague Dawley rats (*n* = 12, 3–5 months old, 136 ± 15 g) were obtained from the Hebrew University Authority of Biological and Biomedical Models. Animals were housed in the animal facilities 3–6 days after delivery for acclimatization and fed ad libitum. On the experimental day, animals were transferred to the lab and anesthetized within one hour of arrival.

### 4.3. Solutions/Media

To improve the health of acute brain slices obtained from mature adult rats, we modified a slice preparation technique optimized for in vitro electrophysiology in mature adult rat brain slices [61,62]. In brief, slice health is improved by a method termed as “protective recovery”. NMDG is used as an extracellular replacer of sodium ions to prevent influx of sodium ions and water into cells as the main insult causing cell death following brain slicing. Ting et al. [62] showed that the presence of NMDG during a 12 min recovery phase after slicing, dramatically improved the health of acute slices from mature adult rats in electrophysiological recordings. Furthermore, incubation in HEPES-buffered artificial cerebrospinal fluid (aCSF) resulted in improved slice health [61]. Altogether, 7 solutions were prepared for each experiment.

Solution 1: The NMDG-aCSF used for the brain perfusion procedure, tissue slicing, and slice recovery contained 93 mM NMDG, 2.5 mM KCl, 1.2 mM NaH_2_PO_4_, 26 mM NaHCO_3_, 20 mM HEPES, 25 mM D-glucose, 10 mM MgSO_4_, 0.5 mM CaCl_2_, 5 mM L-ascorbic acid, 2 mM thiourea, and 3 mM pyruvic acid in double distilled water.

Solution 2: The HEPES-holding aCSF contained 84 mM NaCl, 2.5 mM KCl, 1.2 mM NaH_2_PO_4_, 30 mM NaHCO_3_, 20 mM HEPES, 25 mM D-glucose, 2 mM MgSO_4_, 2 mM CaCl_2_, 5 mM ascorbic acid, 2 mM thiourea, and 3 mM pyruvic acid in double distilled water.

Solution 3: The aCSF used for perfusion in the NMR spectrometer contained 115 mM NaCl, 2.5 mM KCl, 1.2 mM NaH_2_PO_4_, 24 mM NaHCO_3_, 5 mM HEPES, 10 mM D-glucose, 2 mM MgSO_4_, and 2 mM CaCl_2_ in double distilled water containing 10% D_2_O.

All three solutions (1–3) were bubbled with 95% O_2_/5% CO_2_ for at least 1 h prior to use and titrated to a pH of 7.31–7.46 using HCl and/or NaOH.

Solution 4: The TRIS-phosphate dissolution buffer contained 11.2 mM NaH_2_PO_4_, 38.8 mM Na_2_HPO_4_, and 50 mM TRIS. The dissolution buffer was titrated to reach a pH of 7.34–7.44 upon addition of 43 mM pyruvic acid.

Solution 5: A high solvent solution (6x concentrated, i.e., 15 mM KCl, 7.2 mM NaH_2_PO_4_, 60 mM D-glucose, 12 mM MgSO_4_, and 12 mM CaCl_2_) was added to the dissolution buffer after dissolution to bring these solute concentrations in the hyperpolarized medium administered to the slices as close as possible to their concentration in Solution 3.

Solution 6: 6 mL of Solution 4 mixed with 2 mL of Solution 5. The solution was placed in a conical tube at the fringe field of the magnet and was bubbled with 95% O_2_/5% CO_2_ and pre-warmed in a 40 °C water bath.

Solution 7: Solution 6 together with the 4 mL of Solution 4 containing the hyperpolarized [1-^13^C]pyruvate made the hyperpolarized medium that was administered to the brain slices.

### 4.4. Surgery and Slice Handling

Rats were anesthetized using a gas anesthesia system (Somnosuite, Kent Scientific, Torrington, CT, USA). Induction was performed in a chamber with 3.5% isoflurane in room air with a flow rate of 440 mL/min. Following 4–7 min of induction, anesthesia was maintained with 3.1–3.2% isoflurane at the same flow rate. Upon obtaining a negative pedal pain reflex, surgery was initiated. First, the diaphragm was exposed and cut, and the rat was transcardially perfused with 30 mL of ice-cold NMDG-aCSF (Solution 1). The animals were then sacrificed by decapitation, and the brain was rapidly removed and placed in ice-cold NMDG-aCSF (Solution 1). The cerebrum was then cut into four parts (first, a sagittal cut along the hemispheric cleft and then a sagittal cut in the middle of each hemisphere). From each part, 350 μm slices were prepared using a McIlwain tissue chopper (The Mickle Laboratory Engineering Company Ltd., Surrey, UK). The process of brain extraction, from decapitation till the brain was in ice-cold NMDG-aCSF took under 2 min, the slicing procedure was done in less than 10 min. After cutting, the slices were transferred to warm, 32–34 °C, NMDG-aCSF (Solution 1) for 12 min for protective recovery. Then, the slices were transferred to HEPES-holding aCSF (Solution 2) at ~32 °C for 15–40 min incubation before transfer to the NMR spectrometer, where the slices were perfused with the aCSF of Solution 3. Since we found that the amount of slices resulting from an entire rat cerebrum exceeded the upper limit of the active zone of the NMR probe, we separated the brain slices into two approximately evenly sized batches and used only one per experimental day.

### 4.5. Perfusion System and the Administration of Hyperpolarized Medium to Brain Slices

Perfusion of brain slices inside the NMR spectrometer and administration of the hyperpolarized medium to the brain slices were carried out as previously described [46], with a few modifications made in order to support the continuous flow of perfusion media during hyperpolarized injections. Upon transfer to the NMR tube, the brain slices were continuously perfused with aCSF (Solution 3) at a flow rate of 4 mL/min throughout the experiment. 200 mL of this aCSF solution were cycled between a reservoir bottle placed in a 40 °C water bath and the NMR tube. The reservoir was continuously bubbled with humidified 95% O_2_/5% CO_2_ (4 L/min). This bubbling rate maintained a 73.0 ± 6.2% O_2_ saturation in the perfusion medium throughout the time of continuous perfusion (*n* = 9 experiments, Figure S4), determined using an NMR compatible O_2_ sensor (PreSens Precision Sensing GmbH, Regensburg, Germany). Inflow into and outflow from the NMR tube was delivered via medical-grade extension tubes and pumped in a closed circle with a peristaltic pump (Masterflex L/S Analog Pump Systems, Cole-Parmer, IL, USA). The inflow and outflow lines were connected to thin polyether ether ketone (PEEK) lines (inner diameter 0.040”, Upchurch Scientific, Inc., Oak Harbor, WA, USA). The inflow line was located at the bottom of the NMR tube, next to a PEEK spacer, which ensured that the brain slices were held in the sensitive zone of the NMR probe. An NMR compatible temperature sensor was fixed inside the NMR tube, at the level of the center of the probe, for monitoring the temperature throughout the experiment (Osensa, Burnaby, BC, Canada). To prevent the slices from floating to the upper end of the NMR tube, we attached a small cotton patch to the inflow line, approximately 10 cm above the bottom of the NMR tube. One outflow line was placed approximately 12 cm above the bottom of the NMR tube and another back-up outflow line was placed about 14 cm above the bottom. The temperature in the NMR tube inside the spectrometer was calibrated to 33.5–36.3 °C using heated air in the spectrometer and by keeping the inflow lines in a heating blanket at 40 °C until entrance to the NMR bore.

We performed the injections of hyperpolarized [1-^13^C]pyruvate using a bypass constructed of medical-grade tubing and 3-way valves to obtain constant perfusion of the oxygenated hyperpolarized medium which contained solute concentrations that were as close as possible to Solution 3, with the exception of 14 mM of hyperpolarized [1-^13^C]pyruvate (Solution 7). During regular aCSF perfusion (i.e., the time in between injections), the bypass was blocked. For injections, Solution 7 was pressure-injected into the bypass line which was submerged in a 40 °C water bath. The length of the bypass tube was adjusted to allow intake of 10 mL of Solution 7. That is, 2 mL of Solution 7 were ejected into a bin to ensure that the solution which would be pumped into the NMR tube, was freshly oxygenated hyperpolarized medium with appropriate solute concentrations, and without any air bubbles which would interfere with the spectral acquisition. After pressure-filling the bypass line with hyperpolarized medium, the 3-way valves controlling in-flow from the perfusion system to the bypass and out-flow from the bypass into the NMR tube were opened. In this state, the peristaltic pump was able to push the hyperpolarized medium out of the bypass and into the NMR tube containing the brain slices at a constant flow rate of 4 mL/min.

### 4.6. Experimental Workflow

The workflow for each experiment was as follows (Figure 1a). The slices were harvested and placed in the recovery solutions (Solutions 1 and 2, Figure 1a, solid grey line). The slices were then transferred to an NMR tube with circulating perfusion medium, where they were maintained for the remainder of the experiment. Following 0.5 h of recovery in the NMR tube within the spectrometer, ^31^P spectra were acquired for at least 0.6 h for viability and pH monitoring. The first injection of hyperpolarized [1-^13^C]pyruvate was then delivered and ^13^C spectra were acquired during the flow of the hyperpolarized medium through the slices. Following the injection, ^31^P acquisition was resumed. For the ischemia-reperfusion group, the slices were subjected to 11.3 ± 0.3 min (*n* = 6 animals in 6 experimental days) ischemic insult (the perfusion pump was halted), *ca*. 1.5 h following the first injection of the hyperpolarized medium. Immediately after the ischemic insult, a second injection of hyperpolarized [1-^13^C]pyruvate was administered, concomitant with reperfusion (the hyperpolarized medium flow itself provided reperfusion). For the control group there was no ischemic insult prior to the second injection of the hyperpolarized medium. For both groups, a third injection was performed *ca*. 1.5 h after the second injection.

### 4.7. DNP Spin Polarization and Dissolution

Spin polarization and fast dissolution were performed in a dissolution-DNP (dDNP) spin polarizer (HyperSense, Oxford Instruments Molecular Biotools, Oxford, UK) operating at 3.35 T. Microwave frequency of 94.132 GHz was applied for the polarization of a [1-^13^C]pyruvic acid formulation at 1.41–1.50 K. The formulations included 14 mM OX063 radical and 0.7 mM Gd^3+^ (as gadoterate meglumine, Doterm, Guerbet, France). The amount of [1-^13^C]pyruvic acid formulation placed in the polarization cup was 14.48–15.35 mg. Following polarization, this formulation was dissolved in 4 mL of dissolution buffer (Solution 4). The dissolution was ejected into a conical tube which contained Solution 6, using a 6 s of He (g) chase.

### 4.8. NMR Spectroscopy

^31^P and ^13^C NMR spectroscopy were performed using a 5.8 T high resolution NMR spectrometer (RS2D, Mundolsheim, France), equipped with a 10 mm broad-band NMR probe.

#### 4.8.1. ^31^P Spectroscopy

^31^P spectra were acquired with a repetition time of 1.1 s and a flip angle of 50°. Spectra were acquired in batches of 7.6 or 30 min (corresponding to 412 and 1640 excitations, respectively). Six experiments were conducted in each manner. Batches were then combined as needed, as per the ^31^P analysis that was performed.

#### 4.8.2. Hyperpolarized ^13^C spectroscopy

Hyperpolarized ^13^C data were acquired using the product-selective saturating excitations approach [23], applying 2.5 ms cardinal sine (sinc) pulses. Selective excitation for [1-^13^C]lactate and [^13^C]bicarbonate was applied consecutively and repeatedly with 4 s repetition time between pulses, yielding 8 s repetition time for each metabolite. For [1-^13^C]lactate acquisition the selective sinc pulse was centered 214 Hz upfield from the [1-^13^C]lactate resonance frequency, resulting in a [1-^13^C]pyruvate-to-[1-^13^C]lactate excitation ratio of 0.04 (Figure S1). For [^13^C]bicarbonate acquisition the sinc pulse was centered 214 Hz down-field from the [^13^C]bicarbonate resonance frequency (Figure S1), yielding a [1-^13^C]pyruvate-to-[^13^C]bicarbonate excitation ratio of 0.12. Using this selective product excitation, both the [1-^13^C]lactate and the [^13^C]bicarbonate signals were fully sampled upon each excitation and thus nulled prior to the next repetition time.

### 4.9. Calculation of Metabolic Rates

To allow determination of apparent metabolic rates, we used the product-selective saturating excitations approach, as previously described [23]. Briefly, in this way the signal of hyperpolarized metabolites ([1-^13^C]lactate and [^13^C]bicarbonate) is fully sampled and depolarized by each selective pulse, this allows for only newly synthesized metabolites to be detected in the following excitation. The [1-^13^C]pyruvate signal is only minimally excited and is therefore almost not affected by the excitation pulses. To quantify the corresponding metabolite production level, this minimally excited [1-^13^C]pyruvate signal was used as a reference. For quantification we selected a temporal window in which the [1-^13^C]pyruvate concentration was constant and therefore known (14 mM). This selection was based on the flow characteristics of the hyperpolarized medium through the NMR tube (and the slices). As per these characteristics, the [1-^13^C]pyruvate concentration in the NMR tube first increased during the wash-in phase, then reached a plateau at a maximal concentration for at least 80 s, then decreased– during the wash-out phase. To be able to determine the temporal window in which the [1-^13^C]pyruvate concentration was maximal and constant (plateau(, the [1-^13^C]pyruvate signal was corrected for signal decay due to T_1_ relaxation and RF pulsation, using an effective relaxation constant, T_1_eff_, of 57 s (Equation (1)). This T_1_eff_ was chosen experimentally since it was able to correct the [1-^13^C]pyruvate decay curve to display the above flow characteristics for all the injections (a typical injection is shown in Figure 4a).

Equation (1) describes the correction applied to the hyperpolarized [1-^13^C]pyruvate signal to observe these flow characteristics,

(1) $$S_{{\mathrm{pyr}}_{\mathrm{corr}}}\left(t\right)=S_{\mathrm{pyr}}\left(t\right){*e}^{\left(\frac{t}{T1\_eff}\right)}$$

where $S_{{\mathrm{pyr}}_{\mathrm{corr}}}\left(t\right)$, is the corrected hyperpolarized [1-^13^C]pyruvate signal for each time point and $S_{\mathrm{pyr}}\left(t\right)$ is the raw hyperpolarized [1-^13^C]pyruvate signal at each time point. The time points selected for further analysis were those points which comprised the plateau phase of the $S_{\mathrm{pyr}\_\mathrm{corr}}\left(t\right)$ data, determined as no less than 80 % of the maximal point on each injection.

The signals of [1-^13^C]lactate and [^13^C]bicarbonate obtained during this time window were used for the calculation of metabolite production rate using Equation (2),

(2) $$\upsilon_{\mathrm{product}}\left(t\right)=\left[\mathrm{Pyr}\right]*\frac{\rho \times {\mathrm{Vol}}_{pyr}}{\mathrm{TR}}*\frac{S_{\mathrm{pro}}\left(t\right)}{S_{pyr}\left(t\right)}$$

where $\upsilon_{\mathrm{product}}\left(t\right)$ is the production rate of [1-^13^C]lactate or [^13^C]bicarbonate at each time point, [Pyr] is the maximal [1-^13^C]pyruvate concentration that could be delivered (14 mM), ρ is the selective pulse excitation ratio which was 0.04 for [1-^13^C]pyruvate to [1-^13^C]lactate and 0.12 for [1-^13^C]pyruvate to [^13^C]bicarbonate, ${\mathrm{Vol}}_{pyr}$ is the volume occupied by the medium in the sensitive region of the NMR probe (estimated to be 0.5 mL), TR is the excitation interval for each metabolite which is 8 s for both [1-^13^C]lactate and [^13^C]bicarbonate, and $S_{\mathrm{pro}}\left(t\right)$ is the signal of [1-^13^C]lactate or [^13^C]bicarbonate at each time point. Data points with insufficient SNR were excluded.

### 4.10. Determination of Slice ATP Content 

The adenosine triphosphate (ATP) content in the brain slices was determined by integration of the γ-ATP signal observed by thermal equilibrium ^31^P NMR spectroscopy (2060–3296 averages) before each injection, and a comparison to an ATP standard of known concentration (0.1 mol/L) made in house. The γ-ATP signal was corrected for steady-state saturation using a T_1_ of 1.1 s, which was previously determined [63].

### 4.11. Spectral Analysis

Spectral processing and calculation of intensity integrals was performed using MNova (Mestrelab Research, Santiago de Compostela, Spain) and DMFit [64].

### 4.12. pH Determination

The pH was determined using the multiparametric approach described by Lutz et al. [49]. First, the spectra were processed using MNova (Spectral analysis section). The chemical shift was referenced to PCr at −2.5 ppm. The processed spectra were exported and used as the input for the .xlsx file provided by Lutz et al. [49]. The chemical shift range of the Pi signal was manually selected for each spectrum. The .xlsx file can be found in the Supplementary Materials of Lutz et al. [49] in the following link: https://cancerres.aacrjournals.org/highwire/filestream/290125/field_highwire_adjunct_files/0/excelsheet.xlsx. To the best of our knowledge, this is the first time that this approach has been used for the analysis of pH in perfused brain slices.

### 4.13. Statistical Analysis

For each of the groups (ischemia and control), statistical analysis of the variation between the results obtained on the three injections was done using the Kruskal–Wallis single-factor analysis of variance by ranks, treating each injection as an independent group. When a significant difference was found in this test (*p* < 0.05), the non-parametric Tukey-type multiple comparisons using the Nemenyi test was performed [65]. For those comparisons in between injections which were not found to be significantly different on the Kruskal–Wallis single-factor analysis of variance by ranks test, the non-parametric Tukey-type multiple comparison using the Nemenyi test was not performed. Statistical analysis was calculated with Excel (Microsoft, Ra’anana, Israel). Statistical calculations were performed using the textbook bio-statistical formulas for the Kruskal–Wallis single-factor analysis of variance by ranks and for the non-parametric Tukey-type multiple comparison using the Nemenyi test [65].

## Data Availability

The entire dataset is available in the Supplementary Materials.

## Supplementary Materials

The following are available online at https://www.mdpi.com/article/10.3390/metabo11040210/s1, Note S1 and Figure S1: Selective pulse calibration and response, Note S2 and Figure S2: ^31^P spectra during an ischemic insult, Note S3 and Figure S3: ATP content in the brain slices throughout the experiment, Note S4: O_2_ content in the perfusion medium, Table S5: Summary of parameters and data of individual injections included in this study.
