# Supplementary material for: LDH and PDH Activities in the Ischemic Brain and the Effect of Reperfusion—An Ex Vivo MR Study in Rat Brain Slices Using Hyperpolarized [1-13C]Pyruvate
Source: Metabolites. 2021 Mar 30;11(4):210. doi: 10.3390/metabo11040210 (PMC8066106; doi:10.3390/metabo11040210)
Supplement: Supplementary file 1 [file metabolites-11-00210-s001.pdf]

LDH and PDH activities in the ischemic brain and the effect of reperfusion – An *ex vivo* MR study in rat brain slices using hyperpolarized [1-^{13}C]pyruvate

Gal Sapir, David Sahul, Naama Lev-Cohain, Jacob Sosna, J. Moshe Gomori, and Rachel Katz-Brull

Department of Radiology, Hadassah Medical Center, Faculty of Medicine, The Hebrew University of Jerusalem

Supplementary Materials

S1. Selective pulse calibration and response

For the acquisition of ^{13}C spectra following injections of hyperpolarized [1-^{13}C]pyruvate, we used product selective saturating-excitations as previously described [1]. In this technique, the metabolite of interest is fully sampled with each excitation, therefore, only newly synthetized metabolites are detected in the consecutive excitation, while the precursor (*i.e.* [1-^{13}C]pyruvate) is excited to a much lower degree. To quantify the rate of [1-^{13}C]lactate and [^{13}C]bicarbonate formation, their relative response to the selective pulse needs to be characterized. In this study, we used a 2.5 ms cardinal sine (sinc) pulse. The dependence of the signal on the pulse amplitude for on-resonance and for 214 Hz off-resonance excitation is shown in Figure S1. For the on-resonance excitation, the pulse response has the expected sine shape, while for the off-resonance excitation the pulse response reaches a plateau at a pulse of 15-33% of the maximal excitation amplitude in our NMR spectrometer (Figure S1a). We therefore concluded that an effective 90° excitation (*i.e.* the maximal reaction) can be achieved with a range of pulse amplitudes, ensuring the complete depolarization of the metabolite. All calibrations shown below were performed on a standard sample of 40%/60% para-dioxane/deuterobenzene. The pulse profile is shown in Figure S1b and S1c for excitations on [^{13}C]bicarbonate and [1-^{13}C]lactate, respectively. This was done to obtain the ratio of metabolite-to-pyruvate excitation used in the enzymatic rate calculations.

Figure S1. Selective pulse excitation profile. a) The on-resonance (blue) shows a sine-shape behavior. The behavior in 214 Hz off-resonance frequency shows a plateau of maximal excitation at 15-33% amplitude (NMR spectrometer units). Acquisition parameters: 2.5 ms sinc pulse, 4 excitations, 12.5 kHz spectral width, repetition time 90 s, and 16,384 time-domain points. The amplitude was increased in 1% step size (percentage of maximal possible excitation amplitude in our NMR spectrometer).
b) The pulse profile used for the excitation of the [^{13}C]bicarbonate signal. The ratio for the pyruvate-to-bicarbonate excitation was found to be 0.12.
c) The pulse profile used for the excitation of the [1-^{13}C]lactate signal. The ratio for the pyruvate-to-lactate excitation was found to be 0.04.
Acquisition parameters for b) and c): 2.5 ms sinc pulse, 6 excitations, 12.5 kHz spectral width, repetition time 90 s, and 16,384 time-domain points. The maximum intensities of the middle signal of para-dioxane are shown. The offset was increased with a 20 Hz step size.

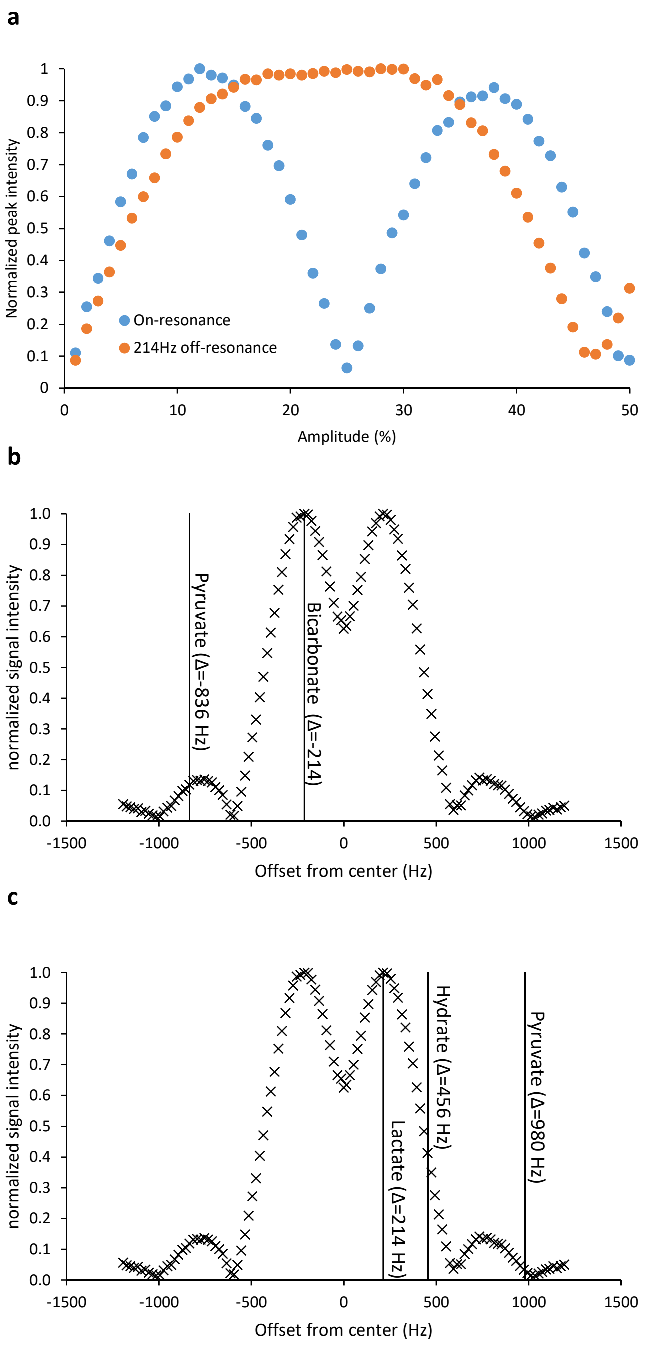

S2. ^{31}P spectra during an ischemic insult

Figure S2 shows ^{31}P spectra acquired during the ischemic insult and immediately following reperfusion. The PCr signal is decreased during the ischemia and increases thereafter. The ATP signals remain largely unchanged immediately during the ischemia but decrease shortly thereafter and then recover. The Pi signal, representing intracellular and extracellular (from the experimental medium) inorganic phosphate, disperses and then narrows. It also increases in intensity due to the large content of Pi in the injection buffer. A "moving sum" of the spectra is shown in Figure S2, such that each spectrum represents 15.1 min of acquisition. This was done to allow better observation of the signals, as the signal-to-noise ratio in spectra acquired over 7.55 min was insufficient.

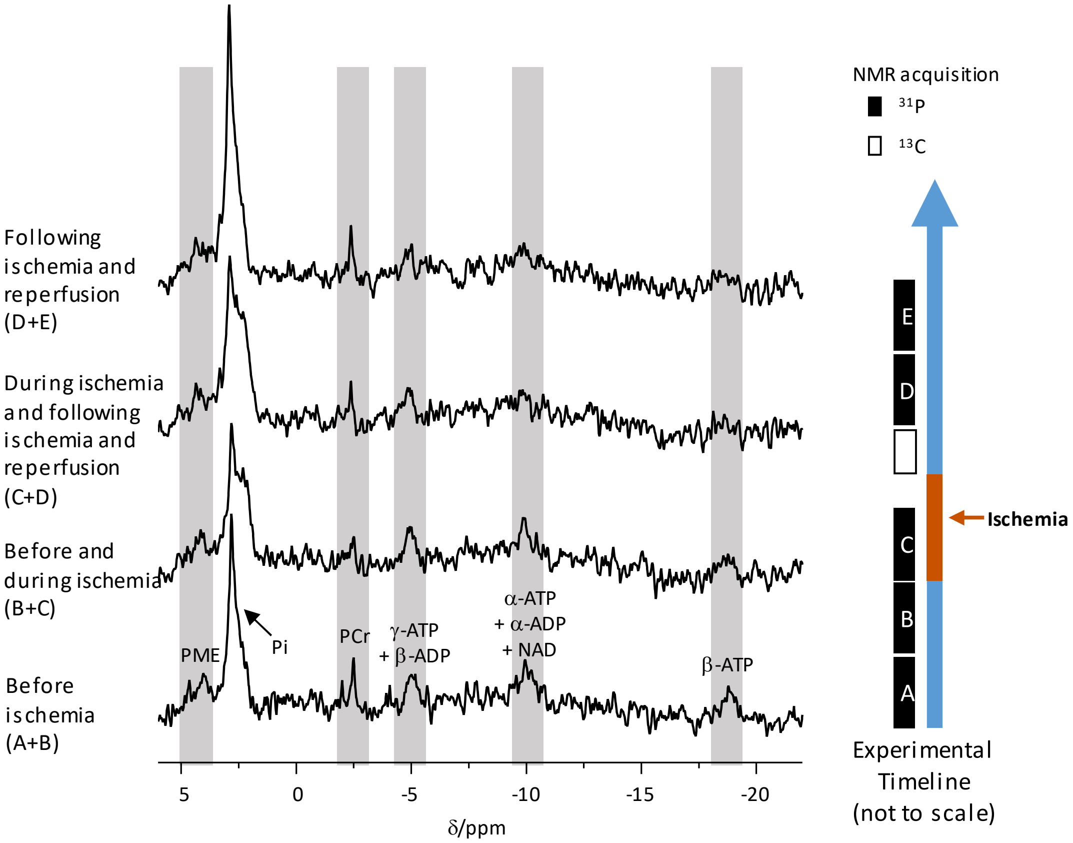

Figure S2. A moving sum of ^{31}P spectra acquired during the experiment.
The temporal acquisition windows (A, B, C *etc.*) for the respective ^{31}P spectra are marked on the right and their combinations are listed on the left. Each spectrum was collected over 15.1 min (824 excitations) in two batches (412 excitations each). The blue arrow in the timeline represents continuous perfusion.
The spectra were acquired using 1.1 s repetition time and a 50° nutation angle, and processed with a line-broadening of 7 Hz and zero-filled from 8,192 to 16,384 points. The spectra were referenced to the PCr signal at -2.5 ppm. ATP – adenosine triphosphate, ADP – adenosine diphosphate, PCr – phosphocreatine, Pi – inorganic phosphate (intra- and extracellular), PME – phosphomonoesters, NAD

- nicotinamide adenine dinucleotide. The shaded areas mark the chemical shift regions of the phosphate metabolites.

S3. ATP content in the brain slices throughout the experiment

Slice ATP content throughout the experiment was determined as described in the Methods. Figure S3 demonstrates a slight decrease in ATP content in the ischemia-reperfusion group. When comparing the ATP content on the time matched groups (before the 1st injection, before the 2nd injection, and before the 3rd injection) we could not find significant differences between the control and the ischemia groups (unpaired two-tailed Student's *t*-test). However, within the ischemia-reperfusion group, between the 1st and the 3rd injection, a significant difference was detected ($p < 0.05$, non-parametric Tukey-type multiple comparison using the Nemenyi test). This difference can be attributed to loss of viable tissue due to the ischemic insult. In the control group there were no differences in the ATP content.

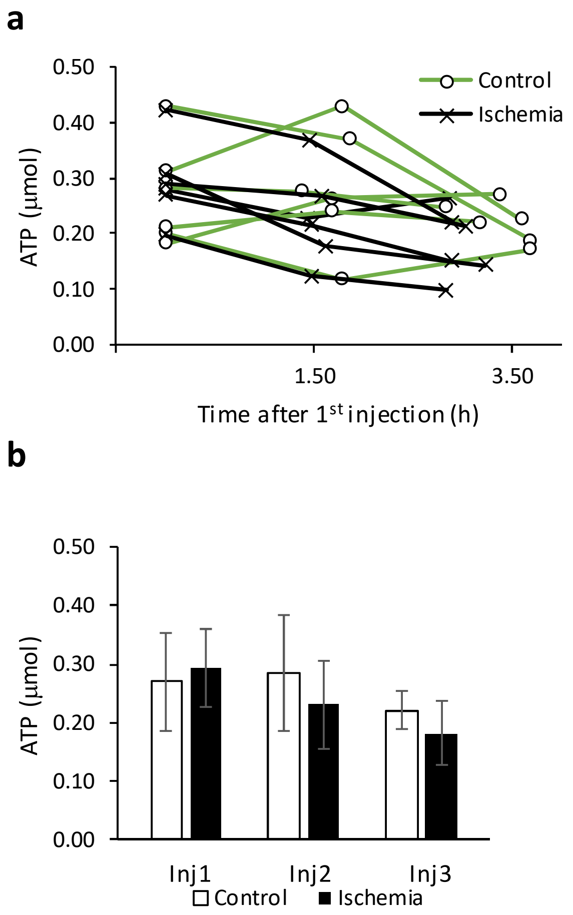

Figure S3. ATP content of brain slices throughout the experiment.
a) ATP content in 3 time points in all the experiments. The spectra used for this analysis were collected immediately before the injections over a time period of 0.6 – 1.0 h (for details, see Supporting Table S5). The time scale is referenced to the end of each acquisition (*i.e.* t = 0 marks the end of the acquisition of the first ATP spectrum, after which the first injection of hyperpolarized [1-^{13}C]pyruvate was performed).

b) ATP content in the two groups. Bars and error bars represent mean and standard deviation, respectively. Inj1 – before the first injection, Inj2 – before the second injection, Inj3 – before the third injection.

S4. O_2 content in the perfusion medium

The O_2 content in the perfusion medium was continuously measured during the experiments using an MR compatible O_2 sensor (PreSens Precision Sensing GmbH, Regensburg, Germany). This was successfully performed in 9 out of the 12 experiments. In the rest of the experiments the measurement was not successful as the readout was corrupt due to contact with the brain slices. In the experiments of the ischemia-reperfusion group, the O_2 saturation was 76.8 ± 3.3 % before the ischemic insult and decreased to 36.3 ± 7.9 % (n = 5 experiments) during the ischemic insult. In a control measurement without brain slices the O_2 saturation was 61.9 % and decreased to 40.5% following the same duration of flow arrest. This difference in relative decrease in O_2 saturation can be attributed to O_2 consumption by the brain slices. To enable this measurement, the O_2 sensor was placed *ca.* 12 cm above the bottom of the NMR tube and thus relatively remote from the brain slices themselves. As a result, the O_2 readings were somewhat delayed, as the media flowed from the bottom of the tube, through the brain slices and then arrived to the O_2 sensor. This likely resulted in an underestimation of the ischemic effect experienced by the slices. At any rate, with this setup, one cannot determine the actual O_2 saturation within the tissue. However, this description provides evidence for the relative decrease in O_2 supply to the brain slices in the ischemia-reperfusion group and the active role of the slices in consuming the available oxygen of the medium engulfing them within the NMR tube during this time.

Table S5. Summary of parameters and data of individual injections included in this study*.

Experiment type	Animal	Injection	^{31}P acquisition time (h)	ATP content before the injection (μmol)	Average LDH activity (nmol/s)	Average PDH activity (nmol/s)
Control	1	1	1	0.43	8.23	0.13
		2	1	0.37	7.38	0.18
		3	1	0.19	8.92	0.08
Control	2	1	1	0.31	11.81	0.25
		2	1	0.43	12.13	0.19
		3	1	0.23	10.62	0.21
Control	3	1	1	0.20	5.92	0
		2	1	0.12	6.09	0.13
		3	1	0.17	5.60	0.16
Ischemia	4	1	1	0.27	9.53	0.19
		2	1	0.22	11.01	0.04
		3	1	0.15	9.57	0.28
Ischemia	5	1	0.6	0.20	12.56	0.43
		2	0.6	0.12	11.87	0.14
		3	0.6	0.10	7.89	0.30
Ischemia	6	1	1	0.31	12.90	0.36
		2	1	0.18	18.50	0.13
		3	1	0.14	10.97	0.28
Control	7	1	0.6	0.21	15.02	0
		2	0.6	0.24	15.59	0.36
		3	0.6	0.22	14.82	0.31
Ischemia	8	1	1	0.29	26.53	0.72
		2	1	0.27	19.42	0.06
		3	1	0.21	12.16	0.24
Control	9	1	1	0.18	19.40	0.26
		2	1	0.26	21.02	0.34
		3	1	0.27	18.28	0.36
Ischemia	10	1	1	0.42	18.19	0.27
		2	1	0.37	23.19	0.06
		3	1	0.22	16.20	0.29
Control	11	1	1	0.28	13.78	0.31
		2	1	0.28	12.56	0.43
		3	1	0.25	9.32	0.36
Ischemia	12	1	1	0.28	11.73	0.35
		2	1	0.23	22.71	0.23
		3	1	0.26	14.13	0.30

*The experiments are listed in the order in which they were actually performed.

References

1 Harris, T. *et al.* Hyperpolarized product selective saturating-excitations for determination of changes in metabolic reaction rates in real-time. *NMR Biomed.* **33**, e4189, doi:10.1002/nbm.4189 (2020).
